# Oncologic Nomogram for Stage I Rectal Cancer to Assist Patient Selection for Adjuvant (Chemo)Radiotherapy Following Local Excision

**DOI:** 10.3389/fonc.2021.632085

**Published:** 2021-03-19

**Authors:** Shutao Zhao, Xin Chen, Dacheng Wen, Chao Zhang, Xudong Wang

**Affiliations:** Department of Gastrointestinal Nutrition and Hernia Surgery, The Second Hospital of Jilin University, Changchun, China

**Keywords:** stage I, rectal cancer, nomogram, prognosis, postoperative adjuvant therapy

## Abstract

**Background:** Because of the low rate of lymph node metastasis in stage I rectal cancer (RC), local resection (LR) can achieve high survival benefits and quality of life. However, the indications for postoperative adjuvant therapy (AT) remain controversial.

**Methods:** A retrospective analysis was performed in 6,486 patients with RC (pT1/T2) using the Surveillance, Epidemiology, and End Results (SEER) database. Patients were initially diagnosed from 2004 to 2016; following LR, 967 received AT and 5,519 did not. Propensity score matching (PSM) was used to balance the confounding factors of the two groups; the Kaplan–Meier method and the log-rank test were used for survival analysis. Cox proportional hazards regression analysis was used to screen independent prognostic factors and build a nomogram on this basis. X-tile software was used to divide the patients into low-, moderate-, and high-risk groups based on the nomogram risk score.

**Results:** Multivariate analysis found that age, sex, race, marital status, tumor size, T stage, and carcinoembryonic antigen (CEA) in the non-AT group were independent prognostic factors for stage I RC and were included in the nomogram prediction model. The C-index of the model was 0.726 (95% CI, 0.689–0.763). We divided the patients into three risk groups according to the nomogram prediction score and found that patients with low and moderate risks did not show an improved prognosis after AT. However, high-risk patients did benefit from AT.

**Conclusion:** The nomogram of this study can effectively predict the prognosis of patients with stage I RC undergoing LR. Our results indicate that high-risk patients should receive AT after LR; AT is not recommended for low-risk patients.

## Introduction

Colorectal cancer is the third most common cancer in the world and the second leading cause of cancer death. While rectal cancer (RC) accounts for one-third of the colorectal cancer cases, most are distal RC (1, 2). In recent years, due to progress with imaging and endoscopy, RC can be detected in the early stage. In the early stage of RC, tumor cells are mostly well-differentiated, the rate of lymph node metastasis is <10%, complete cure can be achieved through local resection (LR), and LR reduces the perioperative complication rate and mortality (3, 4). LR primarily includes transanal resection (TAE) and transanal endoscopic microsurgery (TEM). In 1977, Professor Morson (5) of St. Mark's Hospital in the United Kingdom first published the results of the application of local excision in the treatment of early RC. Only 10 of 119 patients were reported to have a recurrence, and the recurrence rate was 8.4%. Since then, the application of LR in stage I RC has become increasingly widespread.

Studies have shown that risk factors for local recurrence include tumor size > 3 cm, stage > T1, tumor invasion depth of submucosal invasion 3 (SM3) and above, poor differentiation of adenocarcinoma, lymphovascular invasion, and positive margins. However, there is no agreement on risk factors for evaluating recurrence and prognosis, and some studies have shown that age and gender are also high-risk factors for recurrence (6–8). In patients with high-risk factors, the local recurrence rate can reach ~20%, which then requires remedial radical surgery or adjuvant therapy (AT). AT (radiotherapy, chemotherapy, or chemoradiotherapy) can be used as an alternative to remedial radical surgery because it has the potential to not only reduce the recurrence rate and organ-preservation after LR, but also has the same effect on prognosis compared with remedial surgery (9–14). Therefore, this paper also focuses on the clinical effect of AT in patients with RC with a high risk of recurrence after LR.

This study evaluated the prognosis of patients with stage I RC by analyzing various clinical case factors in the Surveillance, Epidemiology, and End Results (SEER) database. The nomogram was used to select candidates for AT.

## Materials and Methods

### Patient Cohort

The SEER^*^Stat (version 8.3.6) software was used to analyze data from 6,486 patients with stage I (pT1/2N0M0) RC diagnosed between 2004 and 2016. Inclusion criteria were: (1) RC confirmed by pathology (ICD-O-3: C20.9); (2) complete follow-up and survival data; (3) adenocarcinoma histology type (ICD-O-3: M-8140); (4) no neoadjuvant radiotherapy received; and (5) completion of LR. The following variables were evaluated: age, sex, race, marital status, histology, tumor grade, tumor size, T stage, carcinoembryonic antigen (CEA), perineural invasion (PI), AT information, and survival information. Cases with unknown information related to these variables were excluded.

### Statistical Analysis

A chi-square test was used to analyze the relationship between the non-AT and AT groups. In order to balance the confounding bias of the included cases, the meaningful clinical pathological factors of the chi-square test were included in propensity score matching (PSM). The nearest neighbor matching was performed at 2:1 in the non-AT and AT groups (15). Then, the Kaplan–Meier method and the log-rank test were used for survival analysis.

In the non-AT group, the prediction model was established by following a series of steps. First, Cox univariate analysis was used to analyze the correlation between variables and overall survival (OS). Second, variables with statistical differences in univariate analysis (*p* < 0.05) were included in the Cox multivariate analysis. Third, on the basis of the Cox multivariate analysis, the nomogram survival prediction model was established. The effectiveness of the prediction model was tested and the degree of discrimination was measured by the concordance index (C-index) (16). The calibration curve intuitively showed the consistency between the predicted survival rate and the actual survival rate, and decision curve analysis (DCA) was used to evaluate the clinical net benefit compared with T stage. Fourth, according to the risk score of the nomogram, X-tile software was used to artificially divide the cases into low-, moderate-, and high-risk groups (17). All statistical analyses in this study were performed using SPSS 24.0 and R language (version 3.6.3), and *p* < 0.05 was considered to be statistically significant.

## Results

### Patient Demographics

**Figure 1:**
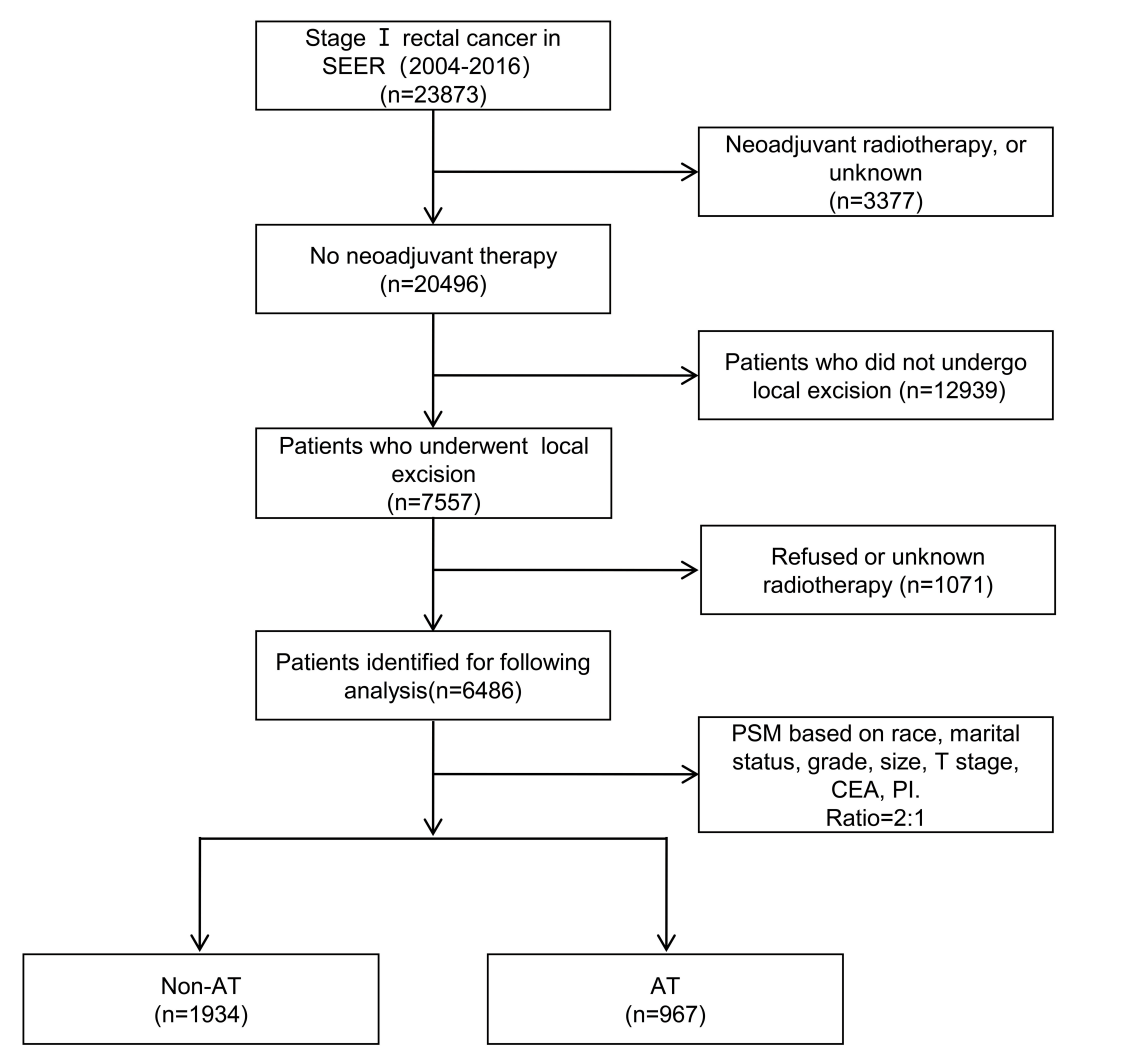
A flowchart of the selection process of included patients.

According to inclusion and exclusion criteria (Figure 1), a total of 6,486 patients were included with LR of stage I RC before the PSM, including 5,519 in the non-AT group and 967 in the AT group. The median survival was 55 months (0–155) and the number of deaths was 2,107 (32.5%). The clinicopathological data showed that AT was significantly correlated with race, marital status, tumor grade, tumor size, T stage, CEA, and PI (*p* < 0.05). After including these variables related to AT for PSM, the final patient number was 2,901, including 1,934 in the non-AT group and 967 in the AT group (Table 1). The median survival in this final cohort was 57 months (0–155) and the number of deaths was 1,098 (37.8%).

**Table 1:** Characteristics of patients.

**Variable**	**Unmatched cohort**			**P-value**	**Matched cohort**			***P*-value**
	**Total [n (%)]**	**Non-AT [n (%)]**	**AT [n (%)]**		**Total [n (%)]**	**Non-AT [n (%)]**	**AT [n (%)]**	
Age				0.227				0.404
<65	2,724 (42.0)	2,335 (42.3)	389 (40.2)		1,136 (39.2)	747 (38.6)	389 (40.2)	
≥65	3,762 (58.0)	3,184 (57.7)	578 (59.8)		1,765 (60.8)	1,187 (61.4)	578 (59.8)	
Sex				0.186				0.137
Male	3,741 (57.7)	3,202 (58.0)	539 (55.7)		1,673 (57.7)	1,134 (58.6)	539 (55.7)	
Female	2,745 (42.3)	2,317 (42.0)	428 (44.3)		1,228 (42.3)	800 (41.4)	428 (44.3)	
Race				0.003				0.987
White	5,286 (81.5)	4,480 (81.2)	806 (83.4)		2,421 (83.5)	1,615 (83.5)	806 (83.4)	
Black	550 (8.5)	458 (8.3)	92 (9.5)		272 (9.4)	180 (9.3)	92 (9.5)	
API	506 (7.8)	446 (8.1)	60 (6.2)		183 (6.3)	123 (6.4)	60 (6.2)	
Other	144 (2.2)	135 (2.4)	9 (0.9)		25 (0.8)	16 (0.8)	9 (0.9)	
Marital status				0.001				0.780
Married	4,056 (62.5)	3,404 (61.7)	652 (67.4)		1,970 (67.9)	1,318 (68.1)	652 (67.4)	
Unmarried	769 (11.9)	656 (11.9)	113 (11.7)		346 (11.9)	233 (12.0)	113 (11.7)	
Unknown	1,661 (25.6)	1,459 (26.4)	202 (20.9)		585 (20.2)	383 (19.8)	202 (20.9)	
Grade				<0.001				0.002
Well/moderately	5,023 (77.4)	4,242 (76.9)	781 (80.8)		2,406 (82.9)	1,625 (84.0)	781 (80.8)	
Poorly/undifferentiated	418 (6.4)	302 (5.5)	116 (12.0)		271 (9.3)	155 (8.0)	116 (12.0)	
Unknown	1,045 (16.2)	975 (17.6)	70 (7.2)		224 (7.8)	154 (8.0)	70 (7.2)	
Size (cm)				<0.001				0.006
<3	2,890 (44.6)	2,394 (43.4)	496 (51.3)		1,587 (54.7)	1,091 (56.4)	496 (51.3)	
≥3	764 (11.8)	552 (10.0)	212 (21.9)		549 (18.9)	337 (17.4)	212 (21.9)	
Unknown	2,832 (43.6)	2,573 (46.6)	259 (26.8)		765 (26.4)	506 (26.2)	259 (26.8)	
T stage				<0.001				<0.001
T1	5,451 (84.1)	4,921 (89.2)	530 (54.8)		1,866 (64.3)	1,336 (69.1)	530 (54.8)	
T2	1,035 (15.9)	598 (10.8)	437 (45.2)		1,035 (35.7)	598 (30.9)	437 (45.2)	
CEA (ng/ml)				<0.001				0.312
≤ 5	1,653 (25.5)	1,336 (24.2)	317 (32.8)		953 (32.9)	636 (32.9)	317 (32.8)	
>5	403 (6.2)	303 (5.5)	100 (10.3)		267 (9.2)	167 (8.6)	100 (10.3)	
Unknown	4,430 (68.3)	3,880 (70.3)	550 (56.9)		1,681 (57.9)	1,131 (58.5)	550 (56.9)	
PI				<0.001				0.280
Negative	2,391 (36.9)	2,082 (37.7)	309 (32.0)		881 (30.4)	572 (29.6)	309 (932.0)	
Positive	34 (0.5)	23 (0.4)	11 (1.1)		27 (0.9)	16 (0.8)	11 (1.1)	
Unknown	4,061 (62.6)	3,414 (61.9)	647 (66.9)		1,993 (68.7)	1,346 (69.6)	647 (66.9)	

*AT, adjuvant therapy; API, Asian/Pacific Islander; CEA, carcinoembryonic antigen; PI, perineural invasion*.

**Figure 2:**
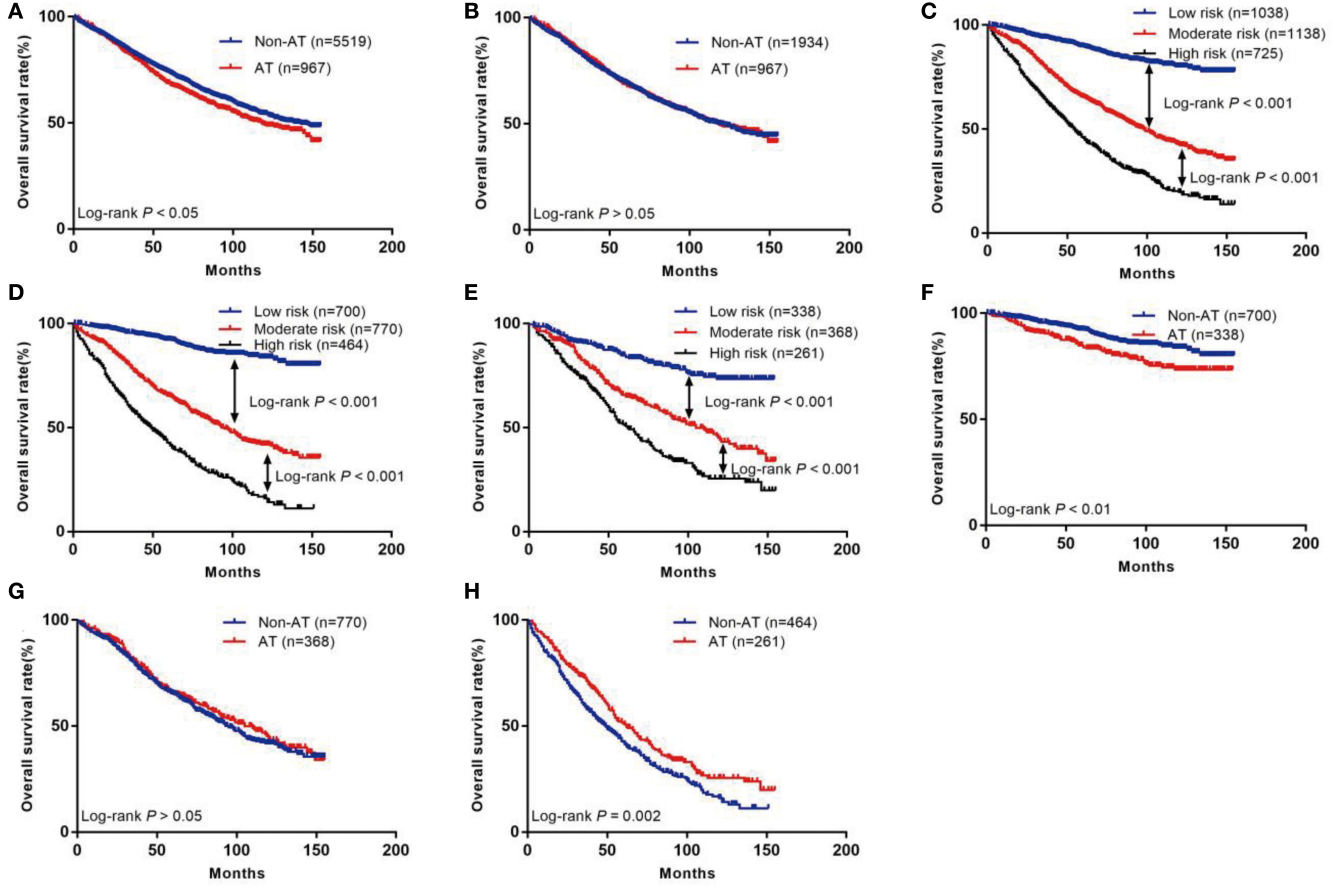
The Kaplan–Meier curves of OS for patients in our study. **(A)** All patients; **(B)** Patients after PSM; **(C)** OS in different subgroups of all patients; **(D)** OS in different subgroups of non-AT group; **(E)** OS in different subgroups of AT group; **(F)** OS for patients with or without AT in low-risk group; **(G)** OS for patients with or without AT in moderate-risk group; **(H)** OS for patients with or without AT in high-risk group.

Before PSM, the prognosis of the group without AT was better than that of the group with AT (5-year survival rate: 73.7 vs. 68.5%; *p* < 0.05; Figure 2A). After PSM, there was no difference in prognosis between the non-AT group and the AT group (5-year survival rate 69.3 vs. 68.5%; *p*> 0.05; Figure 2B).

### Construction of the Nomogram

**Figure 3:**
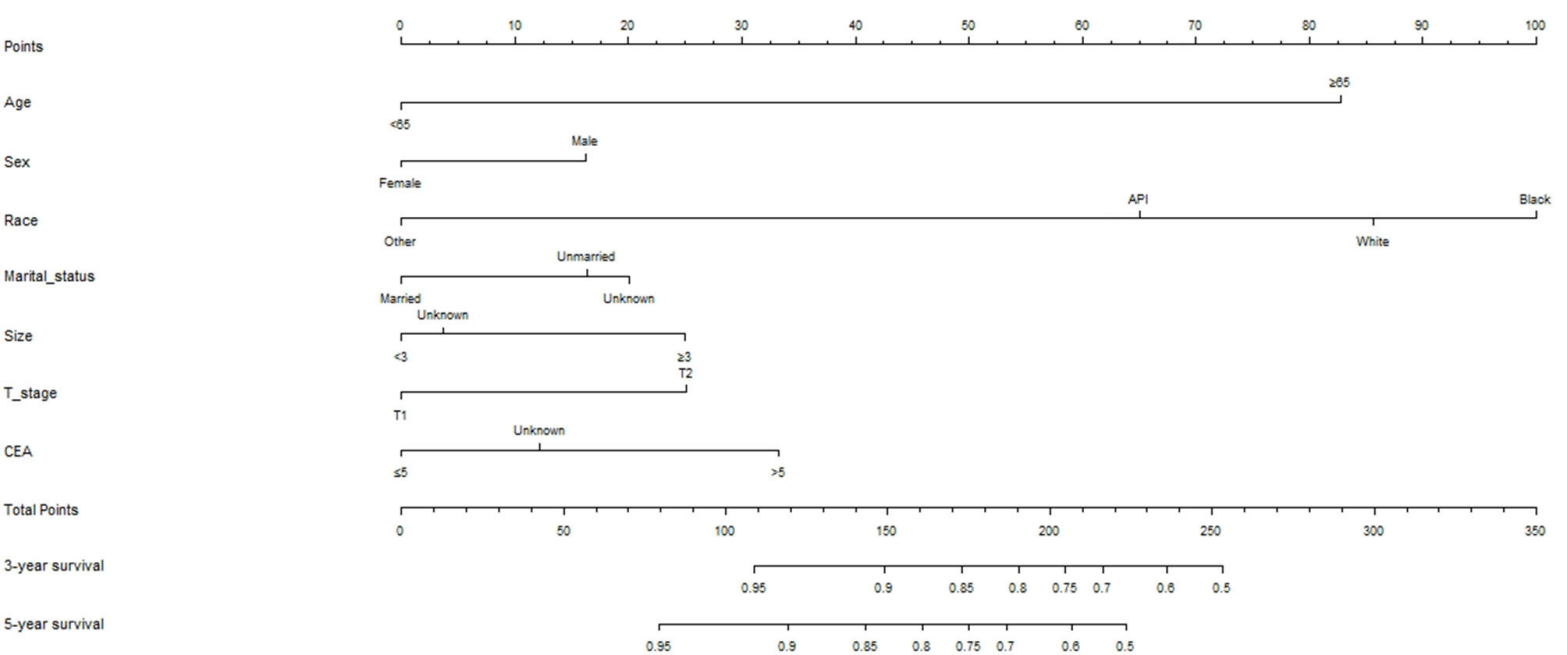
Oncologic nomogram for patients with stage I rectal cancer after local excision.

The data of patients who did not receive AT were included in the Cox proportional hazards regression analysis (Table 2). Univariate analysis showed that age, sex, race, maritime status, tumor grade, tumor size, T stage, and CEA were related to OS (*p* < 0.05). Furthermore, these variables were included in the multivariate analysis, which found that age, sex, race, marital status, tumor size, T stage, and CEA were independent prognostic factors (*p* < 0.05). Based on this, a nomogram was constructed to predict 3-year and 5-year survival after LR of stage I RC (Figure 3).

**Table 2:** The univariate and multivariate analyses of factors associated with overall survival.

**Variable**	**Univariate cox regression**		**Multivariate cox regression**	
	**HR (95% CI)**	***P*-value**	**HR (95% CI)**	***P*-value**
Age				
<65	1			
≥65	5.295 (4.274–6.560)	<0.001	4.446 (3.565–5.545)	<0.001
Sex				
Male	1			
Female	0.854 (0.736–0.992)	0.039	0.747 (0.637–0.876)	<0.001
Race				
White	1			
Black	1.053 (0.817–1.357)	0.691	1.293 (0.998–1.674)	0.052
API	0.672 (0.476–0.950)	0.024	0.690 (0.488–0.976)	0.036
Other	0.136 (0.019–0.970)	0.047	0.180 (0.025–1.281)	0.087
Marital status				
Married	1			
Unmarried	1.122 (0.878–1.434)	0.357	1.342 (1.044–1.724)	0.022
Unknown	2.048 (1.737–2.415)	<0.001	1.434 (1.194–1.721)	<0.001
Grade				
Well/moderately	1			
Poorly/undifferentiated	1.340 (1.047–1.713)	0.020		
Unknown	1.002 (0.758–1.323)	0.991		
Size (cm)				
<3	1			
≥3	1.974 (1.651–2.360)	<0.001	1.568 (1.306–1.881)	<0.001
Unknown	1.070 (0.896–1.278)	0.453	1.069 (0.892–1.281)	0.468
T stage				
T1	1			
T2	2.218 (1.914–2.569)	<0.001	1.572 (1.343–1.840)	<0.001
CEA (ng/ml)				
≤ 5	1			
>5	2.268 (1.768–2.909)	<0.001	1.816 (1.414–2.333)	<0.001
Unknown	1.284 (1.085–1.520)	0.004	1.243 (1.049–1.474)	0.012
PI				
Negative	1			
Positive	1.200 (0.492–2.929)	0.689		
Unknown	1.001 (0.826–1.213)	0.995		

*API, Asian/Pacific Islander; CEA, carcinoembryonic antigen; PI, perineural invasion*.

### Testing the Effectiveness of Predictive Models

**Figure 4:**
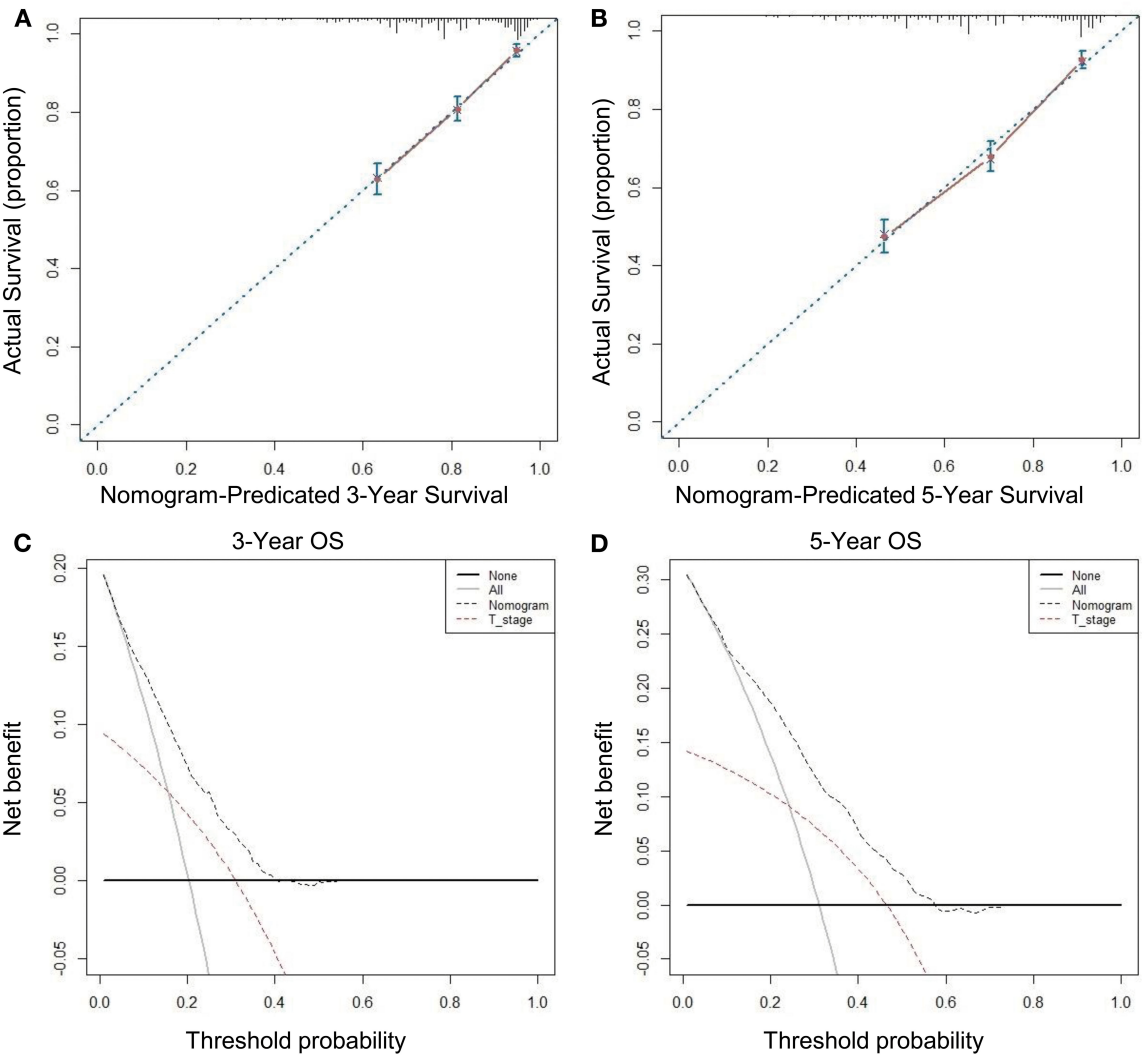
Calibration curves and decision curve for OS prediction: **(A)** 3-year OS calibration curve in our cohort; **(B)** 5-year OS calibration curve in our cohort; **(C)** Nomogram was compared to the T stage in terms of 3-year OS in our decision curve analysis; **(D)** Nomogram was compared to the T stage in terms of 5-year OS in our decision curve analysis.

We used seven variables that were significant upon multivariate analysis to build a nomogram for predicting prognosis. The C-index of the nomogram model was 0.726 (95% CI, 0.689–0.763), which was significantly higher than that of the T stage model 0.594 (95% CI, 0.557–0.631). The nomogram calibration curves of the 3- and 5-year OS indicate that the predicted survival probability was in good agreement with the actual survival probability. DCA was used to determine that the nomogram prognostic model net income for different decision thresholds was higher than the prediction ability of the T stage system (Figure 4).

### Risk Stratification System

**Figure 5:**
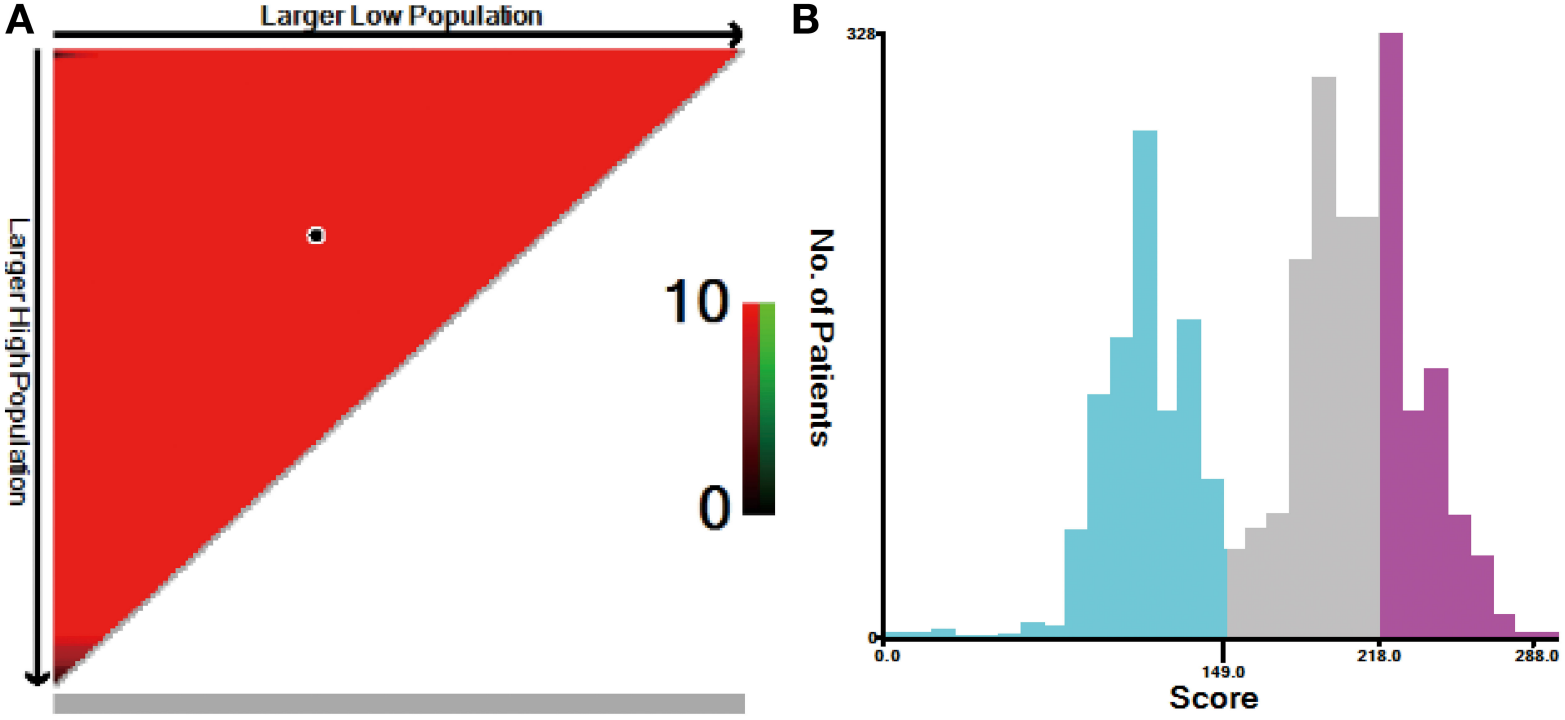
X-tile analysis for risk stratification: **(A)** The optimal cutoff value; **(B)** Numbers of patients in low-, moderate-, and high-risk subgroups.

The risk scores of all patients were calculated using the nomogram (Table 3), and patients were then divided into three risk groups using X-tile software (Figure 5): a low-risk group (score ≤ 149, *n* = 1,038), a moderate-risk group (score 150–218, *n* = 1,138), or a high-risk group (score ≥ 219, *n* = 725). The 5-year survival rates of low-, moderate-, and high-risk groups were 89.7, 65.6, and 46.1%, respectively. The differences were statistically significant (*p* < 0.001, Figure 2C).

**Table 3:** Point assignment of each component and prognostic score for stage I rectal cancer.

**Group**	**Score**	**Estimated 3-y OS (%)**	**Estimated 5-y OS (%)**
Age			
<65	0		
≥65	83		
Sex			
Male	16		
Female	0		
Race			
White	86		
Black	100		
API	65		
Other	0		
Marital status			
Married	0		
Unmarried	16		
Unknown	20		
Size (cm)			
<3	0		
≥3	25		
Unknown	4		
T stage			
T1	0		
T2	25		
CEA (ng/ml)			
≤ 5	0		
>5	33		
Unknown	12		
Total score			
	109	95	
	149	90	
	173	85	
	190	80	
	204	75	
	216	70	
	236	60	
	253	50	
	79		95
	119		90
	143		85
	161		80
	175		75
	187		70
	207		60
	225		50

*API, Asian/Pacific Islander; CEA, carcinoembryonic antigen*.

Through the existing scoring system, we divided the non-AT group into three subgroups: low (*n* = 700), moderate (*n* = 770), or high (*n* = 464). The 5-year OS rates of the low-, moderate-, and high-risk subgroups were 92.3, 65.5, and 42.8%, respectively, with statistical significance (*p* < 0.001, Figure 2D). In the AT group, the 5-year OS rate of the low-, moderate-, and high-risk subgroups was 84.7, 65.8, and 51.8%, respectively, with statistically significant differences (*p* < 0.001, Figure 2E).

### Evaluating the Efficiency of AT for Patients in Different Groups

We further compared the outcomes of low-, moderate-, and high-risk patients receiving AT (Table 4). The results showed that the low-risk group had a poor prognosis after receiving AT (HR = 1.72; 95% CI: 1.21–2.44; *p* < 0.01; Figure 2F), the prognosis of patients in the moderate-risk group receiving AT was similar to that without AT (HR = 0.92; 95% CI: 0.76–1.11; *p* > 0.05; Figure 2G), and patients in the high-risk group benefited from AT (HR = 0.74; 95% CI: 0.61–0.89; *P* = 0.002; Figure 2H).

**Table 4:** Risk stratification in non-AT and AT group.

**Survival status**	**Non-AT Group**				**AT Group**			
	**Low risk [n (%)]**	**Moderate risk [n (%)]**	**High risk [n (%)]**	***P*-value**	**Low risk [n (%)]**	**Moderate risk [n (%)]**	**High risk [n (%)]**	***P*-value**
Live	624 (89.1)	429 (55.7)	156 (33.6)	<0.001	272 (80.5)	215 (84.2)	107 (41.0)	<0.001
Death	76 (10.9)	341 (44.3)	308 (66.4)		66 (19.5)	153 (15.8)	154 (59.0)	

*AT, adjuvant therapy*.

## Discussion

For surgeons, the goal of RC surgery should be to not only radically resect the tumor, but also to maintain the integrity of intestinal and anal functions as much as possible. LR of RC is a surgical method allowing for minimal damage, good oncological effect, and retention of the rectum, and is receiving more attention from clinicians. For patients with cT1N0 rectal cancer without risk factors, the guidelines recommend LR. If found pT > 1, SM3 invasion, poor differentiation, tumor budding, and lymphovascular or perineural invasion, the guidelines recommend follow-up radical resection or AT (18). Borstlap et al. (19) found that patients with pT1/T2 RC who went on to receive AT (*n* = 405) were compared to those who underwent radical resection (*n* = 130) after LR. pT1 RC local recurrence rates for AT and radical resection were 10% (95% CI: 4–21) vs. 6% (95% CI: 3–15), and 15% (95% CI: 11–21) vs. 10% (95% CI: 4–22) for patients with pT2. However, it is important to note that oncology safety is an important factor that restricts the application of this surgical approach. Willett et al. (20) found that the following risk factors contribute to an LR failure rate of more than 20%: tumor size > 3 cm, poor differentiation of adenocarcinoma, lymphovascular invasion, and positive margins. This leads to poor postoperative oncological effects because the presence of these high-risk factors increases the risk of lymph node metastasis. The guidelines for patients with postoperative recurrence risk support recommendation of remedial surgery or AT. However, after LR failure, the highest 5-year survival rate of patients receiving remedial surgery is only 58% (21–23). The latest research shows that AT can achieve the same long-term prognosis as remedial surgery (24). Compared with remedial surgery, AT has advantages in trauma and postoperative complications and can eliminate subclinical lesions so as to improve the local control rate. For patients at high risk for recurrence after LR, AT and follow-up should be given (25). At present, controversies remain about the prognostic factors of stage I RC after LR and the influence of AT on prognosis (26–28). The purpose of our study was to select patients who would benefit from AT after LR.

A better understanding of the high-risk factors for recurrence after LR is of great significance for guiding AT. The incidence of RC among young patients is increasing each year (29, 30). Meyer et al. (9) found that young patients aged 20–39 with T1 stage disease had a worse prognosis than those aged 60–69 years (HR = 1.97; 95% CI: 1.36–2.86; *p* < 0.001). Younger patients aged 20–39 years with T2 stage disease had a worse prognosis than those aged 60–69 years (HR = 1.48; 95% CI: 1.13–1.95; *p* < 0.001). Younger patients with RC were associated with poor tumor cell differentiation, lymphovascular invasion, and a higher rate of distant metastasis than older patients (45 vs. 25%) (31). A study by Patel found that the prognosis of patients with stage I RC aged over 65 years was poor (HR = 2.30; *p* = 0.04) (32). As a result, it is controversial whether old age is a high-risk factor in colorectal cancer. Interestingly, our study found that patients ≥ 65 had a worse prognosis (HR = 5.30; 95% CI: 4.27–6.56; *p* < 0.001). The possible reasons are that the elderly patients in our study had a high proportion of T2 stage disease (39.8 vs. 16.7%) and a high proportion of tumor size ≥ 3 cm (20.8 vs. 12.0%). Furthermore, patients of older age are likely to be in relatively poor physical condition, have more basic diseases, and have a high proportion of postoperative complications (33).

Our study found that female patients had a better prognosis than male patients (HR = 0.75; 95% CI: 0.64–0.88; *p* < 0.001). Yang et al. (34) found that OS (HR = 0.87; 95% CI: 0.85–0.89; *p* < 0.001) and cancer specific survival (CSS) (HR = 0.92; 95% CI: 0.89–0.95; *p* < 0.001) were better in women than in men, which is consistent with our results. Moreover, estrogen in female patients has a positive effect in reducing the incidence rate and mortality of colorectal cancer (35).

Our study also found that blacks had a worse prognosis than whites (HR = 1.29; 95% CI: 1.00–1.67; *P* = 0.052), and the API prognosis was better than that of whites (HR = 0.69; 95% CI: 0.49–0.98; *P* = 0.036), which is consistent with previously published results from Pulte (36). Our research also found that divorced patients have a worse prognosis, which may be related to hormone levels and living conditions. Our study found that tumor size ≥ 3 cm was correlated with a worse prognosis (HR = 1.57; 95% CI: 1.31–1.67; *p* < 0.001), and this is an undisputed high-risk factor for a poor prognosis (20, 37).

It has been reported that the recurrence rate after LR is slightly higher than that after traditional radical resection. The high recurrence rate is mainly concentrated in RC at pT2 stage, while the recurrence rate of RC at pTl stage is not significantly different from that of traditional radical resection (13, 38). The characteristics of lymph drainage vary in different layers of the colon and rectum. There is almost no lymph drainage in the mucosa layer; there is some drainage in the submucosa layer; and most lymph drainage occurs in the muscular layer. Thus, the risk of lymph node metastasis in RC is different depending on the level of invasion of the intestinal wall. The risk of lymph node metastasis is the highest with invasion of the muscular layer. This is the reason for the high recurrence rate and poor prognosis of pT2 RC (39, 40). Indeed, our study also found that patients with pT2 stage RC had a poor prognosis (HR = 1.57; 95% CI: 1.34–1.84; *p* < 0.001).

We know that elevated CEA means that colorectal cancer has a high degree of malignancy and is more likely to have lymphatic or distant metastasis (41). CEA is not considered to be a high-risk factor for recurrence of stage I RC in the National Comprehensive Cancer Network (NCCN) guidelines (42), although our study did find positive CEA to be a high-risk factor (HR = 1.82; 95% CI: 1.41–2.33; *p* < 0.001). With this finding, we further expand the range of risk factors, which is of great significance for a more comprehensive evaluation of patient prognosis.

Moreover, the nomogram that we developed based on these prognostic factors shows good discrimination and repeatability. The C-index of our nomogram is 0.726 (95% CI, 0.689–0.763), which is significantly higher than that of T stage at 0.594 (95% CI, 0.557–0.631), indicating that our nomogram has a stronger predictive ability than the traditional tumor/nodes/metastases (TNM) staging system. We used DCA to further confirm that the nomogram is superior to traditional T staging in predicting the OS of patients with stage I RC.

We introduce this concept in the face of controversy surrounding the influence of AT on the prognosis of stage I RC after LR. The latest review results show that AT is beneficial for high-risk patients in pT1 stage, but has no survival benefit for patients in pT2 stage (26). A study by Jae-Uk found no significant difference in OS between AT and non-AT groups in patients with stage I RC after LR (43), while a study by Wang reported that AT improved OS of pT2 patients (44). The purpose of this portion of our study was to improve the selection of patients who could truly benefit from AT. Our study showed that AT did not bring survival benefits to all patients before and after PSM. This is mainly because AT is often used in clinical patients with already poor prognosis, and therefore beneficial effects are minimal. Therefore, we scored each patient according to their risk factors for recurrence and divided the patients into low-, moderate-, and high-risk groups, so as to accurately treat the target patients. Between the non-AT group and AT group, there were significant survival differences across the three risk levels, which show that our risk stratification is reasonable and effective. In order to investigate which group of patients may benefit from AT, we found that the 5-year survival rate of low-risk patients receiving AT was lower than that of the group not receiving AT (84.7 vs. 92.3%, *p* < 0.01). Therefore, we do not recommend AT for low-risk patients, because our findings suggest that the harm caused by AT outweighed the benefit. The 5-year survival rate of patients at moderate risk who received AT was similar to that of those who did not receive AT (65.8 vs. 65.3%, *p* > 0.05). Therefore, for these patients, consideration to perform AT must take into account all relevant factors. The 5-year survival rate of patients at high risk who received AT was higher than those who did not receive AT (51.8 vs. 42.8%, *p* < 0.01), indicating that high-risk patients are likely to benefit from AT.

This paper comprehensively analyzes the prognostic factors of patients with stage I RC after LR based on the latest large sample data from the SEER database and establishes an accurate and convenient nomogram prognosis model. However, the study is not without limitations. First, the lack of external verification by other populations may reduce the universality of our model. Second, our study is a retrospective study, and the exclusion of some patients with stage I RC due to missing data, or missing risk factors not present in this database could all introduce bias. Third, we do not know the AT regimen and compliance of each patient and the rate of patients with high-risk factors receiving AT and non-AT is different, which will lead to heterogeneity. There is no survival prognostic model incorporated into these clinical pathologic factors for stage I RC after LR. It is most important to stratify patients into different groups, as this has great significance to guide clinical AT. Thus far, there is no conclusion as to whether stage I RC after LR should be observed, AT, or radical surgery, this further highlights the importance of our study. This study analyzed and constructed the nomogram prognostic model based on the SEER large-sample multicenter data, which ensured the robustness of the model.

## Conclusions

Our nomogram effectively predicts the prognosis of stage I RC after LR. AT is recommended for high-risk patients, while AT is not recommended for patients at low or moderate risk.

## Data Availability Statement

The original contributions presented in the study are included in the article/supplementary material, further inquiries can be directed to the corresponding author/s.

## Author Contributions

XW designed the research. SZ took part in designing the research. DW collected the data. XC analyzed the data and wrote the manuscript. CZ collected the data and analyzed the data. All authors approved the final manuscript.

## Conflict of Interest

The authors declare that the research was conducted in the absence of any commercial or financial relationships that could be construed as a potential conflict of interest.
